# Clinical characteristics of patients with Epstein Barr virus in cerebrospinal fluid

**DOI:** 10.1186/1471-2334-11-281

**Published:** 2011-10-21

**Authors:** Timi Martelius, Maija Lappalainen, Maarit Palomäki, Veli-Jukka Anttila

**Affiliations:** 1 Department of Medicine, Division of Infectious Diseases, Helsinki University Central Hospital PO Box 348, 00029 HUS, Helsinki, Finland; 2 Department of Virology and Immunology, Helsinki University Central Hospital, Laboratory Services (HUSLAB), PO Box 400, 00029HUS, Helsinki, Finland; 3 Department of Radiology, Helsinki University Central Hospital, PO Box 340 00029HUS, Helsinki, Finland

## Abstract

**Background:**

The role of Epstein-Barr (EBV) virus in central nervous system (CNS) infections is not fully resolved. It is clearly associated with lymphoproliferative disease of immunosuppressed persons, and may cause encephalitis.

**Methods:**

We reviewed the medical records, imaging and laboratory findings of all patients EBV DNA PCR positive in cerebrospinal fluid (CSF) during 2000 to 2009 in the Helsinki University Central Hospital.

**Results:**

We identified 32 patients with EBV DNA in CSF. 11 had history of allogeneic hematopoietic stem cell transplantation, 7 solid organ transplantation and 5 HIV/AIDS. 5 patients had no preceding immunodeficiency.

In 8 of the cases, another pathogen was identified in CSF. These were *M. tuberculosis *(2), *T. gondii *(2), *Aspergillus *(1), *Herpes simplex virus 1 *(1), *C. neoformans *(1) and *Human herpesvirus 6 *(1). Altogether in 15/32 (47%) of the cases the clinician had a strong suspicion of cause other than EBV for the patients' CNS symptoms/findings.

Of note, 7 of 11 (64%) patients with stem cell transplantation had encephalitis (univariate odds ratio 5.6; confidence Interval 1.1-27.4). Of these 6 had no other pathogen identified.

**Conclusions:**

EBV DNA was often found together with other microbial findings in CSF of immunocompromised patients. EBV seems to be associated with encephalitis in stem cell transplant recipients.

## Background

Epstein-Barr virus infects over 90% of the world population and is the causative agent of infectious mononucleosis. Reactivation of latent EBV is clinically significant in immunocompromised patients and as a rule accompanies lymphoproliferative disease [1].

Central nervous system (CNS) complications of Epstein-Barr virus (EBV) infection occur in 1 to 18% of patients with infectious mononucleosis and include encephalitis, meningitis, cerebellitis, polyradiculomyelitis, transverse myelitis, cranial and peripheral neuropathies, and psychiatric abnormalities [2-4]. EBV is associated with CNS lymphoma and encephalitis, and EBV viral load in CSF is higher than in postinfectious complications [4]. However, this study had a highly selected patient material, e.g. 50% had central nervous system lymphoma.

On the other hand EBV-DNA was commonly detected in CSF of HIV patients with other CNS infections or diseases [5]. In 208 patients with various neurological diseases, among whom the clinician did not request any herpesvirus PCR, no CSF sample was found positive for EBV [6]. Among 253 encephalitis patients studied in France in 2007, only 2,3% had EBV as etiology [7]. However, not all of the patients were tested for EBV.

We wanted to study the clinical significance of CNS EBV DNA positivity in an unselected patient material at a single tertiary care hospital.

## Methods

Positive EBV DNA findings in CSF were identified in the laboratory database of the Helsinki University Central Hospital. Patients with medical records available were included in the study.

We reviewed retrospectively the clinical and laboratory records as well as the radiologist's statements of brain imaging done at the time EBV DNA positivity in CSF.

The clinical diagnosis of encephalitis was based on brain dysfunction (altered mental status with focal neurological signs or symptoms indicative of parenchymal brain involvement) in addition to inflammatory and meningeal symptoms.

The requests for the CSF PCR examinations came from individual clinicians on clinical grounds only, without knowledge of this study.

Underlying disease, stem-cell transplantation, solid organ transplantation and presence of other microbiological findings in central nervous system were recorded.

The study was approved by the ethical and personal data security authorities of the hospital.

### Radiology

The brain MRI studies performed at the time of EBV positivity were retrospectively reanalyzed by one neuroradiologist (M.P.).

### EBV PCR

The DNA was isolated by by using the easyMAG Instrument (bioMérieux). The quantitative real-time EBV PCR assay for cerebrospinal fluid was performed as previously described for plasma samples [8,9]. Briefly, the primers amplifying a conserved sequence of viral DNA polymerase (BALF5) gene and a fluorogenic probe for this area described by Kimura et al. were used at a primer concentration of 0.9 uM and a probe concentration of 0.25 uM. The amplification was carried out in an ABI PRISM 7900 HT Sequence Detector (PE Applied Biosystems) and the standard curve was created with the Sequence Detection System software by plotting the CT values against known EBV-DNA concentrations. The assay has a detection limit of 500 genome equivalents/ml.

### Serology

When available, results of EBV serology including VCA-IgG, IgM and IgG avidity [10] were recorded to assess whether the patient had primary infection or reactivation.

### Statistical analysis

Categorical variables were analysed by cross-tabulation, and Fisher's exact test was used to assess significance. Continuous variables were compared between the groups using Mann-Whitney-U test. All calculations were done with PASW18 (SPSS Inc, Chicago, IL, USA).

## Results

Altogether 322 EBV PCR studies of CSF samples from the Meilahti Hospital of Helsinki University Hospital were performed during 2000-2009.(Table 1). Additional samples from other hospitals were not included in this study.

**Table 1:** Number of CSF EBV PCR tests performed in Meilahti Hospital of Helsinki University Hospital

	CSF EBV PCR tests	Positive CSF samples in study
2000	7	0
2001	11	1
2002	28	6
2003	23	4
2004	41	2
2005	27	0
2006	49	15
2007	52	5
2008	44	10
2009	40	5
Total	322	48

There were 32 patients with one or more EBV DNA positive cerebrospinal fluid samples.

### Most hospital patients with EBV DNA in CSF are immunosuppressed

Of the 32 patients 26 (81%), had an immunosuppressive condition: 11 had history of allogeneic hematopoietic stem cell transplantation, 7 solid organ transplant and 5 HIV/AIDS. In addition 2 patients had hematological malignancy without stem cell transplantation, and one had IgG subclass deficiency. One patient had a suspicion of lymphoma at the time of EBV finding and died of pneumocystis infection and sepsis. 5/32 (16%) patients had no preceding immunodeficiency. Two of these had a clinical picture consistent with mononucleosis and one patient had multiple sclerosis diagnosed at the time of EBV DNA positivity in CSF.

### EBV is commonly found together with other CNS infections

In 8 (25%) of the cases another pathogen was verified in CSF in addition to EBV. These were *M. tuberculosis *(2), *T. gondii *(2), *Aspergillus *(1), *Herpes simplex virus 1 *(1), *C. neoformans *(1) and *Human herpesvirus 6 *(1). Altogether in 15/32 (47%) of the cases the clinician had a strong suspicion of cause other than EBV for the CNS symptoms/findings based on clinical, laboratory, radiological findings, response to therapy, or a microbiological finding in a site other than CNS together with concordant clinical/radiological findings.

In addition one patient developed aspergillus brain abscess 9 months after the EBV (and HSV) positivity, following a relapse of acute mixed lineage leukaemia.

The seven clinically suspected pathogens without microbiological confirmation included tuberculosis/mycobacterial infection in 2 cases, toxoplasma (1) and aspergillus (1), nocardia (one patient, with prior lung nocardiosis and response of CNS lesions to meropenem), JC-virus (one moribund patient with MRI findings typical of PML), VZV (1) and suspected bacterial/fungal infection (1). (Table 2)

**Table 2:** Clinical characteristics of patients with EBV DNA in CSF cerebrospinal fluid

Age	Sex	Underlying disease	Immunocompromise	Central nervous system diagnosis	MRI reanalyzed	Suspected CSF-pathogen	Confirmed other CSF pathogen	encephalitis
65	m	AML	HSCT	encephalitis	Normal			1
50	m	Myeloma	HSCT	encephalitis	Normal			1
60	f	Myelofibrosis	HSCT	encephalitis	Fungus?			1
34	m	Hybrid leukaemia	HSCT	encephalitis		HSV1	HSV1	1
40	m	AML	HSCT	encephalitis	Normal			1
27	m	CML	HSCT	encephalitis	Encephalitis			1
41	f	AML	HSCT	encephalitis	Normal	VZV		1
53	m	MDS	HSCT	mass lesion	Aspergillus	Aspergillus	Aspergillus	0
38	m	CML	HSCT	meningitis	(Polyradiculitis?)	Nocardia	HHV6	0
39	m	ALL	HSCT	PTLD	Infection??			0
58	f	CML	HSCT	pontine myelinolysis	Pontine myelinolysis			0
67	m	Kidney TX	SOT	cerebritis/PTLD	Tuberculosis/lymphoma?	Tuberculosis	Tuberculosis	0
67	f	Kidney TX	SOT	encephalitis/brain abscess	Lymphoma?(tuberculosis?)	Tuberculosis		1
25	m	Kidney TX	SOT	Mass lesion	infection?	Aspergillus		0
53	m	Heart TX	SOT	PTLD	Fungus?			0
63	m	Heart TX	SOT	Mass lesion	Infection or LPD	Bacteria or fungi		0
66	m	Heart TX	SOT	Meningoencephalitis or PTLD?	Lymphoproliferation?(Tuberculosis?)	Tuberculosis		0
45	f	Lung TX	SOT	Meningitis	Normal			0
32	f	HIV	AIDS	Encephalitis	PML	JC-Virus		1
33	m	HIV	AIDS	B-cell lymphoma	Lymphoma			0
47	m	HIV	AIDS	Mass lesion	Toxoplasma	Toxoplasma		0
35	m	HIV	AIDS	Mass lesions	Toxoplasma	Toxoplasma	Toxoplasma	0
48	m	HIV	AIDS	meningitis	Cryptococcal meningitis	Cryptococcus	Cryptococcus	0
64	m	CLL		Mass lesions	Toxoplasma	Toxoplasma	Toxoplasma	0
26	m	Multiple sclerosis	Immunocompetent	Multiple Sclerosis	Multiple Sclerosis			0
33	m	Mononucleosis	Immunocompetent	Mononucleosis+facial paresis	Neuritis			0
52	f	T-cell lymphoma		Lymphoma	Encephalitis??			0
27	m	Tuberculosis	Immunocompetent	meningitis+abscess	Tuberculous meningitis+abscess	Tuberculosis	Tuberculosis	0
56	f	Myeloma		Encephalitis	Infection, toxoplasma?/Fungus?			1
46	f	Ig subclass deficiency		meningitis				0
18	f	Mononucleosis	Immunocompetent	meningoencephalitis	Polyradiculitis?			1
34	m	Viral meningitis	Immunocompetent	encephalitis	Encephalitis? ADEM?			1

HSCT = stem cell transplantation, SOT = solid organ transplant

### Clinical manifestations

In 13 cases clinical diagnosis of encephalitis or meningoencephalitis was made. In 6 patients the clinical diagnosis was meningitis without encephalitis.

**Figure 1:**
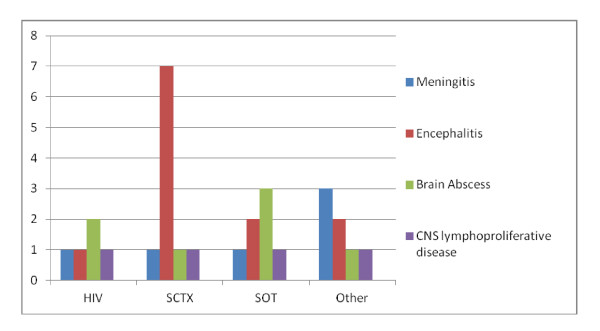
**Clinical manifestations of patients with EBV DNA in CSF**. Y-axis: number of patients. HIV = HIV infection, HSCT = stem cell transplantation, SOT = solid organ transplantation. "Brain abscess" also includes fungal lesions.

Interestingly, 7 of 11 stem cell transplant recipients had encephalitis, compared to 1 of 7 solid organ transplant recipients and 1 of 5 HIV infected patients. The univariate odds ratio for HSCT versus no HSCT to have encephalitis was 5.6 (confidence interval 1.1-27.4) (Figure 1)

In the remaining patients the clinical/radiological diagnoses included brain abscess (tuberculosis, bacteria, fungi, toxoplasma) (7), CNS lymphoma/lymphoproliferative disease (4), mononucleosis with facial paresis (1), multiple sclerosis (1) and pontine myelinolysis (1) (Table 2)

### Imaging studies

30 patients had at least one brain magnetic resonance imaging performed at the time of CSF EBV DNA positivity. For this study our neuroradiologist reviewed the MRI scans in retrospect with similar criteria.

Among the, 7 stem cell transplant patients with encephalitis, 4 had normal MRI at the time of CSF EBV DNA positivity. One had only computed tomography performed initially. One had unenhancing patchy changes both supra- and infratentorially, compatible with encephalitis. Another patient had a hemorrhagic lesion in the cerebellum and small changes supratentorially, suggesting mold infection. Despite broad spectrum antibiotics and voriconazole the patient recovered only after rituximab.

In the 7 patients with solid organ transplantation, only one had normal MRI. In 5 patients there were single or multiple contrast enhancing lesions, and in 4 of these neuroradiologist suggested lymphoproliferative disease or infection (tuberculosis in 3 cases, of whom one was confirmed microbiologically, and also the remaining 2 received empirical antituberculous therapy). One patient withmultiple edematous lesions with hemorrhages and enhancement, suggesting fungus, responded clinically to voriconazole. In addition one patient with unenhancing lesions considered in retrospect suggestive of fungus, had disseminated PTLD, with a brain biopsy positive for EBV encoded RNA (EBER).

None of the five HIV patients had normal MRI: one had findings compatible with B-cell lymphoma, two had toxoplasma lesions, one had PML, and one a small focal change compatible with cryptococcal meningitis.

Three previously healthy patients had varied changes compatible with the clinical picture: contrast enhancement of facial nerves in facial paresis following mononucleosis in one patient, acute disseminated encephalomyelitis in another and lesions in cerebellar peduncles and radices in a patient with meningoencephalitis followed by polyradiculitis.

### CSF cells and chemistry

**Figure 2:**
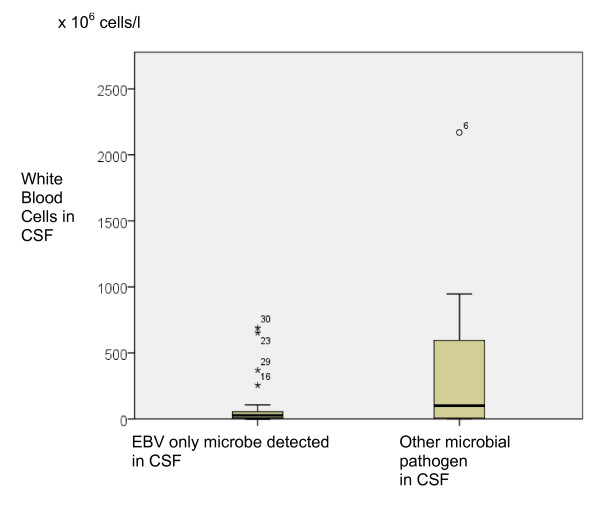
**Number of leukocytes in CSF (× 10^6 ^cells/liter) Leukocyte number in cerebrospinal fluids with EBV only or with other pathogens (difference not significant)**. Box plots show the median, interquartile range, error bars and outliers.

The median amount of white blood cells and protein in CSF was higher in the patients with an additional pathogen. However, the variation was great and the difference not significant (Figure 2).

### Viral load in CSF

A quantitative real time PCR was used in the study, but since the quantitation was at the time only validated for plasma samples, the clinician received the result as either positive or negative. Viral load should here be regarded as semi-quantitative. Retrospectively, a CSF viral load was available for 8 of the 32 patients. The copy number ranged from 186 to 76,800 copies/ml. The highest copy number occurred in a PTLD patient.

### Viral load in plasma

EBV PCR in plasma was examined in 28 patients, in 22 within one week of the CSF sample. The plasma PCR at any time was positive in 14 patients.. The plasma viral loads ranged from 530 to 1,040,000 copies/ml. with the highest copy numbers again found in the PTLD patients.

In patients with EBV as the sole CNS pathogen (found or suspected), 9 of 14 had positive plasma EBV PCR, compared to 4 of 13 patients with other concomitant CNS infections (OR 4.05, CI 0.85-19.35).

### Serology

EBV antibodies had been studied in 25 of the 32 patients. Only 2 had positive IgM and low IgG avidity indicating primary EBV infection. These were previously immunocompetent patients with mononucleosis. Two more had borderline IgM. One of these was a solid organ transplant recipient with persistently negative EBV IgG and prolonged viremia, diagnosed later with EBER-positive PTLD.

The remaining patients were EBV IgG positive and IgM negative indicating existing EBV immunity and reactivation rather than primary infection.

### Outcome

Ten patients died during the follow-up. with a median survival time of 41 days (range 5 to 1073). All four patients with malignant CNS lymphoproliferative disease died. Two patients died of aspergillosis.

However, only one of the 7 stem cell transplant patients with clinical encephalitis died during the follow-up. The deceased patient died 348 days after observed CSF EBV positivity, due to disseminated (including CNS) aspergillosis. The patient also had HSV1 DNA positivity concomitant with EBV DNA. Therefore the prognosis of EBV encephalitis in stem cell transplant recipients seems to be relatively favorable.

## Discussion

In this study we observed EBV DNA mainly in the CSF of severely immunocompromised patients with central nervous system disease. This finding is consistent with earlier studies [4] with majority of patients having an immunosuppressive condition.

In addition to EBV in 8 cases another pathogen was found in CSF, and in further 7 cases the clinician finally suspected other etiology based on radiological, biochemical or clinical features or response to therapy. In 17 of our 32 patients EBV was the only pathogen found or suspected. Of these 17 patients, 7 had encephalitis, 3 meningitis and 4 CNS lymphoproliferative disease.

In another study coinfection with another pathogen was found in 25% of cases with EBV DNA in the CNS [11] with evidence of EBV replication in the CNS.

In miscellanous neurological patients CNS "colonization" with EBV seems to be rare [6,7]. However, in infectious mononucleosis the CSF is often positive for EBV.

Our (and others')cohort is probably heavily biased by the clinicians propensity to look for EBV in CNS mainly in profoundly immunocompromised patients.

Most of our patients had serology consistent with past EBV immunity and reactivation. This could imply that other infections in the CNS may trigger EBV reactivation. Surprisingly, EBV DNA was found in CSF also in the absence of plasma EBV DNA. Unfortunately this was not consistently examined in this patient material. In many cases there was delay between the CSF and plasma samples, which could explain the negative plasma PCR in cases of transient viremia. An intriguing alternative explanation is the preferential EBV replication in the CSF possibly stimulated by other CNS infections. Technically, there should be no difference in the sensitivity of the assay. Clinicians should be encouraged to order simultaneous plasma EBV assay whenever studying EBV DNA from the CSF.

Unfortunately, at present no simple way seems to exist to determine whether EBV is the cause of an individual patient's CNS disease. Exclusion of other pathogens, absence of brain abscess-like lesions (with the caveat of lymphoma) and high concomitant plasma level of EBV DNA, and perhaps history of allogeneic stem cell transplantation in the context of encephalitis can support a primary role for EBV.

Inpatients with EBV DNA in the CSF a consideration of reduction of immunosuppression, if feasible, is warranted. Rituximab is well established in treatment of EBV associated lymphoproliferative disease [12]. However its role in EBV-associated encephalitis is unclear. The same is true for antiviral agents and i.v. immunoglobulin.

Multiplex PCR tests for simultaneous detection of several herpesviruses are used increasingly. This will shed more light on the real frequency of the EBV presence in the CNS of patients hospitalised due to neurological symptoms, when also previously healthy individuals will be tested.

Indications to test CSF for EBV DNA include suspicion of CNS PTLD or lymphoma in solid organ- and stem cell transplant patients and HIV patients. Encephalitis in stem cell transplant patients propably warrants EBV testing of CSF, among other tests. In other patient groups the indications are less clear but in general, profound deficiency of T-cell mediated immunity predisposes to complications of EBV infection.

However, even in these patients, CSF EBV PCR should be interpreted with caution, and meticulously exclude other, treatable microbial pathogens.

More studies are needed that include tissue level demonstration of EBV to demonstrate the pathogenic role of EBV in CNS diseases other than PTLD.

## Conclusions

In immunocompromised patients with EBV DNA in CSF other microbial pathogens often coexist. Especially in HSCT patients EBV appears to be a significant CNS pathogen.

## Competing interests

The authors declare that they have no competing interests.

## Authors' contributions

All authors read and approved the final manuscript. TM contributed to study design, reviewed the clinical data, did the analyses and wrote the paper. ML was responsible for the virological methodology. MP reviewed the radiological exams. VJA designed the study and contributed to article writing

## Pre-publication history

The pre-publication history for this paper can be accessed here:

http://www.biomedcentral.com/1471-2334/11/281/prepub
